# Integrity links ethics and efficiency in nursing leadership: Nurse leaders’ views

**DOI:** 10.1177/09697330251374397

**Published:** 2025-09-05

**Authors:** Susanne Salmela, Jessica Hemberg

**Affiliations:** 1 Unit of Research and Development, Vaasa Central Hospital, Vaasa, Finland; 2 Department of Health Sciences, Faculty of Science and Engineering, 65100 Åbo Akademi University, Vaasa, Finland

**Keywords:** caring, ethics, health care, moral perspectives, nursing administration, nursing management

## Abstract

**Background:**

Today’s healthcare organizations are organized to provide effective, efficient care that yields good patient outcomes. Healthcare leaders must be capable of engaging in both leadership relevant to human relationships and the management of processes.

**Aim:**

The aim of this study was to explore nurse leaders’ perceptions of integrity in enhancing nursing leadership that is both effective and ethical.

**Research design, participants, and research context:**

A qualitative exploratory design with qualitative content analysis as method was undertaken, including multistage focus group interviews with 9 nurse leaders in a hospital setting.

**Ethical considerations:**

The study was conducted in accordance with the guidelines of the Finnish National Board on Research Integrity and the Declaration of Helsinki.

**Findings:**

One main category and three subcategories were seen. The main category was “Integrity links ethics and efficiency”.

**Discussion:**

Integrity was seen as a part of ethical leadership. Integrity was also seen as nurse leaders’ values shaped by personality and work experience. To lead with efficiency was seen as part of knowledge-based leadership.

**Conclusions:**

Integrity allows nurse leaders to interweave ethical values and efficiency, thereby realizing nursing leadership that is ethical and effective and leading to care that is of good quality.

## Introduction

Today’s healthcare organizations are organized to provide effective yet efficient care that yields good patient outcomes and creates value for patients. Effectiveness in healthcare refers to the delivery of care that achieves desired health outcomes and improves patient well-being,^1^ while efficiency is defined as providing healthcare services in a manner that maximizes resource use and minimizes waste, delays, and unnecessary costs.^2^ Healthcare organizations are dependent on information relevant to patients, care, and health^3^ to ensure the availability and development of excellent-quality healthcare. Healthcare leaders must be capable of engaging in both leadership relevant to human relationships and the management of processes^4,5^ the so-called “managerial leadership” according to us. This kind of dual competency enables leaders to balance interpersonal effectiveness with operational control, which is essential for influencing outcomes in complex healthcare systems. In order to manage human relationships healthcare leaders need to use ethical leadership, which is defined as leading with integrity, fairness, and accountability while promoting ethical standards, transparency, and respect for patients and staff.^6,7^ Such leaders foster trust and moral decision-making to ensure high-quality, patient-centered care.^6,7^ Change leadership is also needed to improve care and operational processes.^8^

Several different concepts can be used to describe the different aspects of efficiency. For example, target efficiency can be defined as *the degree of achievement of objectives in relation to the use of resources*,^9^ that is the administering of available resources in such a way that set goals can be achieved. Target efficiency can be considered well suited for use in the public sector, where financial gain is not the primary goal.^10^ When seeking to provide respectful, dignified, and safe care that is also evidence-based, financially viable, and sustainable,^11^ nurse leaders must balance many different demands. The tension that arises from seeking to balance competing values and meet both quality and efficiency requirements can result in nurse leaders feeling that their integrity is being compromised.^12^ Integrity within healthcare leadership is defined as the consistent alignment of actions with ethical principles, fostering trust, transparency, and accountability in decision-making.^5^

Leaders who lead with integrity are present, visible, and actively engaged both as team leader and as part of the team.^13^ As seen in an integrated literature review, integrity plays a major role in strengthening effective management.^14^ While integrity in a care context is most often associated with patients, nurse leaders’ integrity is associated with ethical practices, actions, and underlying meanings^15^ and can be compared to leading with integrity. Integrity is often understood to have both personal and moral significance^12^ yet is a complex concept that encompasses many different aspects.^16^ Integrity can be viewed as both a virtue and a quality^17^ and can be interpreted in ethical leadership as nurse leaders’ honesty and uprightness. Nurse leaders’ honesty and uprightness refers to consistently behaving with transparency, truthfulness, and moral integrity—even under pressure—to serve as role models and build trust among their teams.^18^ Integrity can also be considered an important aspect of ethical leadership through which the effectiveness of a care leader can be explained.^19^

### Background

In this background section, effective and ethical nursing leadership is outlined, along with how integrity is linked to it. Through their work, nurse leaders support compassionate and safe patient care^20^ and play a crucial role in cultivating positive work cultures.^21^ Effective leaders are inspirational and charismatic and have integrity and regard for others’ needs.^22^ Integrity can be considered the heart of leadership.^23^

In our study, the concept effective is defined in relation to nursing leadership and nurse leaders, while efficient is considered an umbrella concept that encompasses the concepts contribution and productivity.^9^ The concept integrity is defined in relation to the ethical leader, leaders with a strong, unwavering morale who demonstrate ethical values in their surroundings through behavior. Ethical values in healthcare are defined as the moral principles—such as autonomy, beneficence, non-maleficence, justice, confidentiality, integrity, and trust—that govern behavior and decision-making in clinical practice and institutional policies.^24^ Integrity is linked to identity, bearing responsibility, and retaining one’s character in one’s commitments, that is, through values and ideals.^25^ Integrity also encompasses critical reflection (making professional judgments based on own ethical values), accountability, and responsibility. Responsibility in healthcare is about doing the work (task-oriented),^26^ while accountability is about owning the outcomes (result-oriented).^27^ In healthcare leadership, accountability—the obligation to be answerable for decisions and outcomes affecting patient care and organizational performance—is crucial,^27^ while responsibility—the duty to competently carry out assigned tasks and roles that contribute to those outcomes—is equally essential.^26^ Integrity is further built upon dialog and the enabling of a personal, professional, organizational, and interorganizational responsibility.^28^ Organizational processes and hierarchical management can negatively impact integrity and morally “courageous” behavior in nursing.^29^

Regarding leadership traits, integrity is the key for leaders and relates to being committed to and brave in doing the right thing for the right reason, regardless of the circumstances and even when no one else is looking.^30^ Leaders can be said to “walk the talk” regarding values and ethics when their actions, methods, principles, and beliefs are based on their fundamental values. Nonetheless, to maintain consistency, a willingness to adjust values or framework when challenged is also needed. Leaders with high levels of integrity leave their egos behind, have sound principles, motivate through ethical behavior, and act with honor and truthfulness. They value empowerment, transparency, and a high level of teamwork and are in a constant learning mode. Through own behavior and interaction, good leaders motivate others to achieve organizational goals. Also, irrespective of other positive characteristics, people will follow leaders who are respectful and responsive and who they trust.

In nursing care, integrity strengthens efficient nurse leadership when it is interwoven into nurse leaders’ leadership and management.^28^ Integrity also strengthens nurse leaders’ ethical approach, that is, the manner in which they lead relationships, processes, and cultures in an organization. To lead relationships means to trust, foster open communication, and promote collaboration among healthcare team members.^11^

To ensure efficiency and quality assurance, it is vital that nurse leaders use different leadership styles, establish a team spirit, and mobilize and coordinate other healthcare professionals. Nurse leaders who lead with integrity are good managers and demonstrate good morals that suffuse their core values and personal and professional principles. This also shapes nurse leaders’ authenticity and courage, and courage is needed for admitting one’s own mistakes and shortcomings as a leader. When nurse leaders lead with integrity, an organization’s care culture, values, and ethical standards and the behavior of those professionals working within the organization can be shaped. To this end, nurse leaders can use strategic planning, the coordination of interrelated care processes, quality assurance, and efficient performances. Integrity seen as interrelated sets of standards and personal, professional, and organizational level values also impact nurse leaders’ decisions and behavior.^11^

Effective leaders and managers must hold many threads in their hands to (proactively) react to the changing conditions that exist within nursing care.^31^ Effective nurse leaders must manage, adapt, and lead through constant change while also managing and improving the quality of care. Thus, nurse leaders must not only manage both financial and human resources but they must also communicate effectively, delegate, work in teams, evaluate performances, and influence organizational structures.

Effective leaders are even task-, relationship-, and change-oriented.^12^ As seen in one model of leading change, change is achieved when leaders lead processes, relationships, and the culture of an organization.^32,33^ While nurse leaders are impacted by the culture of an organization, they also influence the culture.^12^ To uphold care efficiency and sustainability, nurse leaders create contextual, professional, and cultural conditions to preserve not only the core and art of caring but also the ethical and professional competence of other nursing staff.^32^

Effective leadership and leading with efficiency^34^ cannot be fully described without also describing knowledge-based leadership, because they are closely related. Knowledge-based leadership in healthcare refers to the ability of leaders to acquire, apply, and share evidence-based and experiential knowledge to support informed decision-making, improve organizational performance, and enhance patient care.^35,36^

Comprised of different components, knowledge-based leadership includes leaders’ skills and their ability to collaborate effectively.^37^ They further highlight that effective collaboration is based on open and transparent communication channels through which all an organization’s many elements can exchange ideas and experiences through regular dialogues and where the voice of all is respected.^37^ Knowledge-based leadership also entails the innovation and integration of knowledge, that is, the utilization of advanced integration methods and practices to reach organizational goals.^38^

While both patient integrity^39^ and ethical leadership in healthcare^40^ have previously been researched, there currently exists scant research on simultaneously leading with efficiency and integrity in relation to nurse leaders.

### Theoretical perspective

Leading with integrity within nursing care is associated with ethical approaches and actions, which is emphasized within the caritative caring theory of Eriksson.^41,42^ In this theory, the main mission of caring is to alleviate suffering by maintaining dignity through love and compassion. The caritas motive is the essential value of caring and which implies a spontaneous and altruistic willingness to take responsibility for and sacrifice something for the other.^41^ Caring, according to Eriksson,^41^ is the core of nursing care and the caring communion is the foundation. Caring communion refers to a deep, ethical, and spiritual connection between the caregiver and the patient, characterized by mutual presence, respect, and openness; it is a sacred interaction rooted in love and charity, where both individuals meet as whole human beings.^42^ Love is unconditional concern for the other, motivating compassion and empathy, while charity (caritas) is the expression of this love through caring actions (it is love in action), forming the ethical foundation of caring and guiding healthcare professionals to alleviate suffering with compassion and respect for human dignity in healthcare.^42^ Ethical values are the basis for a caring culture within an organization^39^ and the patient is always placed in the center and should be encountered with respect and sensitivity to the patient’s vulnerability in order to safeguard his or her dignity.^40^ A caring culture is defined as an organizational environment where the values of compassion, dignity, love, and responsibility guide leadership, interpersonal relationships, and care practices.^43^ Life values are highlighted in the caritative leadership, and the understanding that every co-worker and patient are unique and their respective life situations. Ethical values and attitudes toward others are thus emphasized and caritative leadership has the potential to enable the creation of a caring culture together with co-workers.^43^ Compassion, communion, and responsibility are essential in a caring culture, which includes bearing responsibility for oneself and others.^42–44^ Ethics according to Salmela ^32^ is reflected in the nursing care that nurse leaders provide, and it can be seen in their willingness to serve, but also in their way of being as a person, and in their relationships with patients and co-workers. Ethics is thus essential for caritative caring and good and high-qualitative care.

## Aim

The aim of this study was to explore nurse leaders’ perceptions of integrity in enhancing nursing leadership that is both effective and ethical. The research questions are as follows: (1) What are nurse leaders’ perceptions of leading with integrity? (2) What are nurse leaders’ perceptions of leading with effectiveness?

## Methodological aspects

A qualitative exploratory design with qualitative content analysis as method^44^ was undertaken, including multistage focus group interviews with 9 nurse leaders.

### Data material, collection, and analysis

Twelve nurse leaders from somatic inpatient and outpatient units in a hospital setting in Finland were invited to participate in focus group interviews, with 9 in total ultimately participating. Those asked to participate were selected in consultation with the Chief Nursing Officer and the Directors of Nursing Care at the study setting.

The participants were all female, aged 54–63, and had five or more years of work experience. All 30 credits in leadership or master’s degree in administration (one) except for one have had 15 credits in leadership. The participants were divided into two groups, with four in group 1 and five in group 2. Multistage focus group interviews inspired by Hummelvoll^45^ were conducted in the summer of 2019, lasting about 2 h per group. A first follow-up interview was conducted in the summer of 2020 with seven participants, followed by a second follow-up interview a few weeks later with six participants.

The interview questions were sent to participants prior to start of the focus group interviews. The research questions and text material containing interpreted results from the focus group interviews were sent to participants prior to the first follow-up interview. Updated text material was sent to participants prior to the second follow-up interview. Participants were allowed to comment on the text material received and provide clarifications and/or further feedback during both follow-up interviews.

Transcription and inductive analysis of the focus group interview material (text material 1) took place in autumn 2019. Transcription and analysis of the first follow-up interview material (text material 2) took place in summer 2020, and the second follow-up interview material (text material 3) autumn 2020.

The interview material was analyzed using qualitative content analysis according to Graneheim and Lundman.^44^ Emanating from the study’s purpose and questions, both researchers read the material several times while looking for meaningful content and relevant categories. The resulting meaning units were then condensed and categorized into one main category and four subcategories, presented below.

### Ethical considerations

The guidelines for good scientific practice as described by the Finnish Advisory Board on Research Integrity, TENK,^46^ and the Declaration of Helsinki^47^ were followed. Ethical approval for the study was obtained from the health care organization in which the research was conducted. Permission to conduct the study was also granted by the Chief Nursing Officer of the same organization, where participant interviews took place. All participants received both written and verbal information outlining the study’s purpose, procedures, and data protection measures, and each provided written informed consent prior to participation. Participation was entirely voluntary, and participants were free to withdraw at any stage without consequence. All data were stored securely on an external hard drive, kept in a locked facility, and protected by encryption, with access restricted exclusively to the research team.

## Findings

The main category was “Integrity links ethics and efficiency.” The three subcategories were “Integrity as part of nurse leaders’ ethical leadership,” “Integrity as part of nurse leaders’ values shaped by personality and work experience,” and “Leading with efficiency as part of knowledge-based leadership.” In the following, we use NL as an abbreviation for nurse leader.

### Integrity links ethics and efficiency

We found that integrity is a key link between ethics and efficiency in healthcare leadership. Without integrity, nurse leaders cannot apply ethics and efficiency in a high-quality manner. Thus, integrity is integral to ethical leadership because it enables nurse leaders to intertwine ethical values with efficiency, thereby providing high-quality nursing care. Integrity also reveals the different levels of leadership, that is, who nurse leaders are leading. The different levels of leadership mean that nurse leaders may include themselves, their group (where the group’s structure and culture influence the leader’s role), and the individuals within the group, as unique leadership is required to meet each person’s needs. *In nursing leadership, ethics and efficiency are linked together through integrity…* (NL 4).

### Integrity as part of nurse leaders’ ethical leadership

According to the participants, when nurse leaders are authentic, meaning honest, true to themselves, and true to their way of leading they act in an honest and upright or principled manner, which facilitates their leading with integrity and thereby ethical leadership. *You can’t play a false role; co-workers see through it. You must truthfully be yourself* (NL 1). Integrity as a part of ethical leadership entails being true and acting in an honest and upright manner, for example, engaging in open communication, having the courage to speak up or set boundaries, or accepting criticism in a professional and non-personal manner. *When you have made a decision, you stand by it, that is, that you are upright* (NL 1). *Even ethically important to set boundaries*... *that now we can no longer do more things efficiently*... *to dare to set the boundary as well... So that you know and can tell when the boundary is crossed so you know when it is still safe*... (NL 7). *It takes a lot of humility, to realize your faults!* (NL 2).

The participants also perceive that integrity as a part of nurse leaders’ ethical leadership is linked to trust of their nurses. Nurse leaders’ integrity is furthermore strengthened through reflection. The participants note that reflection can include reflection on own or organizational values or even professional aims. *Sometimes you need to stop and think “why am I here and what is my task?” Sometimes I can use this to lead ethically... to think why we are here*... (NL 2).

The participants also describe how experiences and challenges shape nurse leaders’ integrity, and thereby their ethical leadership, and can even bolster nurse leaders’ resolve to act with integrity. *Challenges [are] instructive because then integrity is also formed through trials and crises*. *Even a lot of courage is needed*... (NL 2).

### Integrity as part of nurse leaders’ values shaped by personality and work experience

According to the participants, integrity encompasses the ethical values and moral principles that are integrated into nurse leaders’ actions and interactions with other employees, healthcare professionals, and the organization itself. The participants describe how integrity serves as a guiding principle or common thread for nurse leaders, enabling the purpose and goal of care work to be anchored in core values and creating a foundation for the decisions that nurse leaders make. *My personal integrity is based on what I myself value, and I value honesty. I value equality and I highly value kindness and even charity... From within my own values comes [the] strength to lead with quality* (NL 3).

The participants perceive that the strength to lead with integrity is linked to nurse leaders’ inner values and can facilitate understanding that acting in a fair manner is not always so easy. *Fairness doesn’t always mean it’s the same thing*... *Fairness is not always the black and white*... *We often discuss this with the work staff... [To act in a fair manner]*... *for the organization and for the employee*... (NL 6). The participants also highlight that integrity is connected to being true to own values and leading oneself. *Integrity serves as a guiding [principle], a common thread.* ... *Behind every decision so I can always go back to my purpose*... *the purpose and goal of the work, why I’m at work* (NL 1). *It’s important to lead yourself, self-leadership: think about whether you’ve made the right decision from an objective point of view* (NL 4).

Integrity for the participants is also associated with trusting that other healthcare professionals, including one’s own nurses, are fulfilling their responsibilities. *I have such an integrity that I trust my co-workers, I don’t intend to look over anyone’s shoulder [regarding] what they’re doing instead I tell them that they can do what they want in between as long as they get [the] tasks [they’re responsible for] done* (NL 3).

The participants even emphasize that leading with integrity in nursing care involves “soft” values that must be balanced against the organization’s “hard” values, which are governed by, for example, financial and legal issues. The participants note that soft values are especially important in a nursing context because such a context is intimately concerned with other human beings.

We can’t just go the legal route... We are people who care for people... so then we have values and soft values that guide us... and that’s the way it must be... and that’s why it becomes so difficult and complex... otherwise it would be easy if you only would allow yourself to be guided by legal decisions, if it was only hard values that directed (NL 3).

According to the participants, NLs’ integrity is shaped during childhood and influenced by both personality and work experience, meaning that it is formed partly by their individual character and partly by their previous professional experiences. *The integrity that [forms] the bedrock has been shaped from your childhood, from your childhood home*... (NL 3). *Personality comes into play when you should lead with integrity*... *but simultaneously you must think that you have a professional role*... *the personal may still not emerge, but instead you must think that you still can act in an objective manner*... (NL 5).

However, the participants highlight that personal viewpoints should not and must not control NLs’ actions, that is, NLs must remain unbiased in their professional role. The participants moreover perceive that NLs must remain objective and set personal boundaries. *It’s important to even set your personal boundaries, toward the group, because otherwise you won’t be able to cope in the job in the long run*. *The group even influences how the leader develops and how his or her integrity is formed* (NL 2).

The participants also observe that integrating integrity into decision-making allows NLs to make well thought-out decisions. *It’s important to not make decisions too quickly… listen to several different [perspectives] before you make decisions. It’s not worth listening to the loudest ones* (NL 4). The participants even perceive that work experience helps shape NLs’ integrity. *All work experience has shaped one as well, so you gain a secure foundation to stand on... it is probably very important* (NL 2).

### Leading with efficiency as part of knowledge-based leadership

The participants describe that leading with efficiency requires that NLs must be perceived as trustworthy, which they link to knowledge-based leadership that encompasses both expertise and an evidence-based approach. The participants also highlight that knowledge-based leadership enables NLs to justify and clarify the basis for the decisions they make. *Right now this is what we know… so therefore right now we do this!* (NL 1).

The participants perceive that to support others NLs should act as effective nurse leaders and engage in effective leadership, including reflecting on and understanding what needs to be done, organizing and prioritizing work duties/tasks, and finding the right person for the right task. This occurs through the ability to understand the different competencies of the healthcare professionals included in their group. The participants also note that collaboration is a part of effective leadership. ...*in order to lead with efficiency the NL needs to have the group with her*... (NL 2).

The participants observe that because a working group affects NLs’ ability to lead, effective leadership requires that the entire working group is “seen,” that is, leadership must be shaped outgoing from the group if it is to be effective. The participants furthermore note that it is important for NLs to be actively present on their unit or within their department. *That you are actively responsive and actively available*... *and so that you can take problems right away so they don’t get out of hand.... and sort out... Discussing leadership... present in the department*... (NL 4).

The participants also note that the ability to identify various team members’ particular skills or competences and, for example, select the right person for the right task, is essential to effective leadership. *The group influences the leader’s manner and also [the leader’s] possibilities to lead, otherwise it will not be an effective leadership if you do not take notice of the group’s composition, weaknesses, and strengths among the staff*... (NL 3).

The participants perceive that in order to realize the organization’s goals while simultaneously focusing on the core of caring, NLs should “steer,” organize, and realize actions and activities so that the right things are performed in the right way. The participants describe how this can take the form of NLs sharing their leadership (shared leadership) or delegating specific tasks/duties to those who may have better knowledge or more experience. *Effective leadership is about successfully sharing the work/task with the person who has the strength and/or knowledge that is needed* (NL 1). *You also need to bounce things [off] others, and work in pairs, and use each other’s strengths as a part of effective leadership. You need to talk to the [appropriate] mentor, and the unit nurses should even discuss between themselves* (NL 8).

The participants even reveal that it is important that NLs understand from which perspective they should lead and are capable of adapting their leadership to each unique situation. *Do you lead the staff through the [tasks/duties] or do you lead the [tasks/duties] through the staff... what is most important…and everything of course overlaps* (NL 9).

The participants note that to maintain efficiency NLs must have the knowledge to make ethical decisions and prioritizations. Nevertheless, the participants simultaneously highlight that hard values must not take precedence when or if pressure for efficiency originates from outside an organization or the organization itself. They furthermore stress that NLs must have the courage to set boundaries regarding demands for efficiency, that is, NLs should have the knowledge and understanding to “say stop” when increased efficiency is no longer possible. *I was forced to take a surprisingly hard line all the time [during the COVID-19 pandemic*] (NL 2). *It’s about thinking ethically that what are we really doing... that we actually need all of this... Are we doing the right thing or can we leave something out...? Staff must learn to ethically prioritize... To prioritize what to do if it’s urgent... What to leave out and what not to do if it’s urgent... So that it doesn’t hurt the patients much* (NL 6).

## Discussion

In this study, we aimed to explore nurse leaders’ perceptions of integrity in enhancing nursing leadership that is both effective and ethical. We found that integrity links efficiency and ethics through nurse leaders’ ethical (leadership) and knowledge-based leadership. In this study, integrity as part of ethical leadership in nursing leadership entails leading with integrity, is seen by the nurse leaders as knowing and acting in accordance with one’s ethical values, and where integrity serves as a guiding principle. This is in line with previous research where integrity implies the presence of ethical practices and actions within nursing care context.^15^ Integrity as an aspect of ethical leadership also plays an important role in strengthening (the ability) to lead with efficiency^15,19,34^ in nursing care. This is also in line with the caritative caring theory which implies that ethics should permeate caring^41,42^ and that the nurse leader together with co-workers cultivate a caring culture which entails ethical values^43^ such as having compassion, communion, and responsibility for oneself and others.^48^

Leading with integrity means, according to the nurse leaders in this study, being true to oneself, honest, and upright (principled manner) including their manner of leading, or in other words leading themselves, (human) relationships, and processes as well as trusting the staff in a nursing care context. This is comparable to previous research in which good leaders are found to be honest and genuine^17^ and true to their own decisions^25^ in their way of leading.^14^ Also Orvik^12^ states that integrity is seen as having both a personal and a moral significance. Another aspect of leading with integrity in nursing care in this study is having courage, setting boundaries, and having an open communication, as well as being able to accept criticism. In line with our findings, Numminen et al.^49^ stress that courage entails an action involving ethical conviction, determination, and persistence and even defending what is believed to be “correct” and maintaining one’s ethical position. It also includes to be committed to do the right thing for the right reason, even when it is difficult or unpopular^28^ because the integrity of the nurse leader is his or her morale and sense of responsibility which is the basis for leading with effectiveness,^12,25,40^ which means using resources in an optimal way.^9^ However, both integrity and courageous behavior can be negatively affected by organizational processes and hierarchical management structure.^29^

We discerned that integrity, according to our participants, is a characteristic trait of nurse leaders, encompassing ethical values and moral principles. These were seen to be integrated into nurse leaders’ actions and interactions with the organization, employees, and other healthcare professionals. Moreover, we found that integrity is linked to personality and work experience from work life and the nursing context. Personal viewpoints should not and must not control nurse leaders’ actions, according to the participants in our study. As seen in previous research, leaders with strong integrity can strengthen an entire organization by attracting competent employees, because people prefer to work for leaders with strong integrity.^30^ Still, we found, according to our participants, that nurse leaders must remain unbiased and objective in their leadership role and even set personal boundaries. Leaders with high levels of integrity leave their egos behind, have sound principles, motivate through ethical behavior, and act with honor and truthfulness.^30^

Integrity is, according to the findings in this study, strengthened through reflection: on own or organizational values or even professional aims within nursing care. According to Robinson and Doody,^28^ integrity includes self-reflection on one’s ethical values and responsibility for making the right decisions as a nurse leader. The nurse leader who leads with integrity help to shape the virtues of authenticity and courage and possesses a good moral foundation that encompasses their foundational values and personal and professional principles, according to the participants in this study. Previous research has described integrity in nursing leadership as the combination of both personal and professional integrity in a dialog and as a portal to authentic leadership, within which human relationships form the foundation.^49^ Other research has also found that integrity is built on dialog and by making the way for a personal, professional, organizational, and interorganizational responsibility.^28^

We found that leading with integrity entails nurse leaders’ balancing soft and hard values, meaning ethical aspects and financial and legal issues. In line with this, the caritative leadership according to Bondas^43^ also emphasize that ethical values need to be considered by the nurse leader. When seeking to provide a respectful, dignified, and safe care that is also evidence-based, financially viable, and sustainable,^11^ nurse leaders must balance many different demands. The tension that arises from seeking to balance competing values and meet both quality and efficiency requirements can result in nurse leaders feeling that their integrity is being compromised within nursing care.^12^

In healthcare, integrity is essential to lead with efficiency.^14^ This means that the nurse leader needs to have the staff with them and have an awareness of what to do and prioritize, as well as know how the work should be organized within nursing care. An integrity in which leadership and management are interwoven strengthens nursing care and results in effective nurse leadership.^14^ Collaboration is an important part of effective leadership and entails, for example, basing one’s leadership on the requirements of the group in nursing care. The establishment of a team spirit and the mobilization and coordination of healthcare professionals is also instrumental to efficiency and quality assurance.^14^

To preserve effectiveness in nursing care the nurse leaders should create contextual, professional, and cultural conditions.^11^ Nonetheless, as seen in our findings, it is essential that hard values are not prioritized over soft values when leading with efficiency, since soft values can preserve patient quality, such as safety and dignity. Hemberg et al.^40^ also found that safeguarding the dignity of the patient was essential for high-quality care. Hemberg and Salmela^14^ highlighted that nurse leaders who lead with integrity are good managers and demonstrate good morals that use their core values and personal and professional principles. This also means that nurse leaders should be proactive,^31^ change-oriented,^19^ taking into account relationships, processes, and culture within nursing care.^32,33^

Leading with effectiveness is, as previously stated, linked to knowledge-based leadership, that is, to have, among other things, knowledge about which skills or competences are needed on different kinds of units in the organization. It is also highlighted in previous research that performance effectiveness and efficiency is^50,51^ linked to knowledge, to achieve their goals and to optimize the use of human resources.^34,52^ This is in line with another definition of knowledge management, in which knowledge not only improves efficiency and performance it also increases patient safety, reduces costs, and facilitates the organization of knowledge and organizational learning.^32^ As such, knowledge management should hold a significant role in the implementation of various processes, for example, the coordination and integration of services^32^ in a change-oriented way by the effective leader^19^ by leading, relationships, processes, and culture in leading change or implementation.^32,33^

We moreover found that leading with efficiency requires to make well-founded decisions and set boundaries that nurse leaders should be perceived as being credible and can justify their decisions and set boundaries regarding demands that could be detrimental to staff, patients, and operations. Nurse leaders must use their integrity to make informed and/or knowledge-based decisions that thereby support nurse leaders leading with efficiency in the meaning of the efforts that nurse leader makes to achieve both good patient outcomes and efficiency in healthcare^9^ (p. 61), which is essential for safe patient care.^53^

### Strengths and limitations

One strength is that both researchers participated equally in the study, conducting individual analysis before together discussing the final analysis and writing the manuscript together. Another strength is that the participants assessed, revised (for clarity), and validated the study findings during a final meeting. One limitation is that one of the focus group interviews was conducted during the COVID-19 pandemic, which may have contributed to the low number of participants in the final meeting and may also have influenced the focus of the study findings.

## Conclusion

Integrity was found to link ethics and efficiency in nursing leadership by both ethical and knowledge-based leadership. Nurse leaders are constantly challenged to lead with efficiency by delivering high-quality care with limited resources. Integrity is thereby essential to leading with efficiency, as ethical decision-making ensures that resources are used wisely without compromising patient safety or trust. Integrity allows nurse leaders to interweave ethical values and efficiency, thereby realizing nursing leadership that is ethical and effective and leading to care that is of good. Integrity needs to be a central focus for healthcare organizations that aim to develop competent, ethical leaders. A focus on the exploration of integrity in nursing leadership as seen from nurses’ perspectives should be included in future research.
